# Use of chinese and western over-the-counter medications in Hong Kong

**DOI:** 10.1186/1749-8546-5-41

**Published:** 2010-12-10

**Authors:** Vincent Chi Ho Chung, Chun Hong Lau, Frank Wan Kin Chan, Joyce Hoi Sze You, Eliza Lai Yi Wong, Eng Kiong Yeoh, Sian Meryl Griffiths

**Affiliations:** 1 School of Public Health and Primary Care, The Chinese University of Hong Kong, Shatin, Hong Kong, China; 2 School of Pharmacy, The Chinese University of Hong Kong, Shatin, Hong Kong, China

## Abstract

Benefits of engaging community pharmacists in providing wider primary care are internationally acknowledged; in Hong Kong, however, strategies for harnessing their potential contributions are yet to be launched. Here, community pharmacist and Chinese medicine retailers are responsible for providing western and Chinese over-the-counter (OTC) medications. Patterns of OTC uses reflect the characteristics of populations who rely on community pharmacists and Chinese medicine retailers as their main point of contact with the healthcare system. Analyzing the data from a Hong Kong survey (*n *= 33,263) on self medication and medical consultation patterns, we propose, in this article, an extended role for community pharmacists and Chinese medicine retailers, which entails aspects as follows: (1) referring patients to other medical services where appropriate; (2) providing health education and preventative services; (3) safeguarding the use of Chinese herbal medicines.

## Background

In Hong Kong, community pharmacists work independently from medical doctors who often prescribe and dispense medications in a clinical setting. On the other hand, patients often seek first line treatment from community pharmacists [1]. Community pharmacists have long been an underutilized part of the human resources in primary care [2,3] as a result of the interplay between demand, supply and organization factors [4]. In Hong Kong, the use of over-the-counter (OTC) medications is popular in the local population. Previous studies found that 65% of the respondents used OTC medications [5] and that 32.9% of outpatients had taken OTC two weeks prior to their visits [6]. The majority of community pharmacists in Hong Kong admitted that they were most frequently asked about OTC [7].

Chinese OTC medications are used as often as their western medicine counterparts in Hong Kong [8]. Unlike pharmacists, tertiary education is not a prerequisite for retailing Chinese medicine OTC [9]. Historically, Chinese medicine retailers worked alongside with Chinese medicine practitioners [10]. Since 1997, Chinese medicine practitioners as a medical profession have been recognized [11] and have become less dependent on Chinese medicine retailers.

This article describes the behavioral patterns of both Chinese and western medical consultations and OTC use in a representative sample of the Hong Kong population. This information will provide timely input for planning pharmacists' and Chinese medicine retailers' future roles within the Hong Kong primary care system [12].

## Data from Thematic Household Survey

Thematic Household Survey (THS) was conducted between November 2005 and March 2006 by the Census and Statistic Department (CSD), Hong Kong [13]. The THS covered the entire land-based population of Hong Kong and interviewed a total of 33,263 non-institutional individuals (response rate: 79.2%). The interviews were conducted in Cantonese. The sample represents a population of 6,750,652 persons of the general population.

## Survey questionnaire on the use of OTC medications

**Figure 1:**
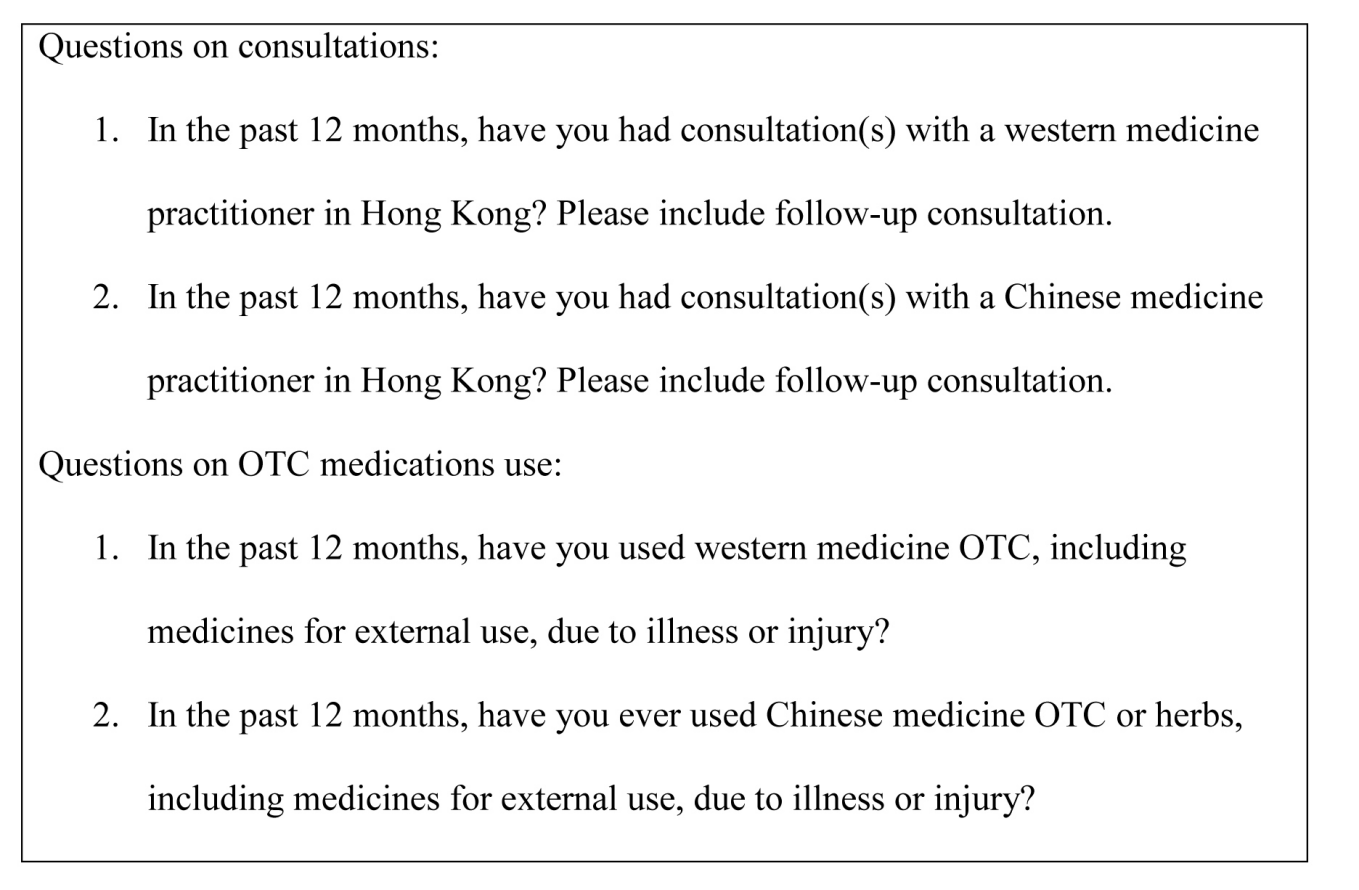
**Questions on consultation and OTC medications use in the THS**.

The questionnaire of THS included a part to solicit information from respondents aged 14 or above on their consultations with western medicine practitioners or Chinese medicine practitioners, as well as their use of western or Chinese OTC medications the past 12 months (Figure 1).

Questions about demographic, socioeconomic and health related information were also covered in the THS. These included gender, age, martial status, personal monthly income, education level, self reported chronic disease status as informed by a western medicine practitioner and self perceived level of health and possession of western or Chinese medicine insurance coverage.

## Our data analysis

Analysis of THS data were conducted without imputation of missing data. We focused our multivariate data analysis on respondents who either consulted a western/Chinese medicine practitioner or used OTC medications in the past year (*n *= 13,346). Sample characteristics were described by cross tabulations of the three patterns (OTC use only, sought consultations only or both) with other demographic, socioeconomic and health related variables. Chi square and one way ANOVA tests were conducted. Multinomial logistic regression analyses were conducted with various demographic, socioeconomic and health related factors as independent variables and patterns of OTC and medical service use as dependent variables. Dependent variables were classified as 'OTC use only' and 'using both OTC and medical services' while 'medical consultation only' was used as a reference. The regression analyses provided adjusted odd ratios for each independent variable, representing its association with the choice of 'OTC only' or 'using both OTC and medical services'. All statistical analyses were performed with SPSS 14.0 (SPSS Inc., Chicago, IL, USA), separately for western and Chinese medicine.

## Use of western OTC medications and consultations with western medicine practitioners

**Figure 2:**
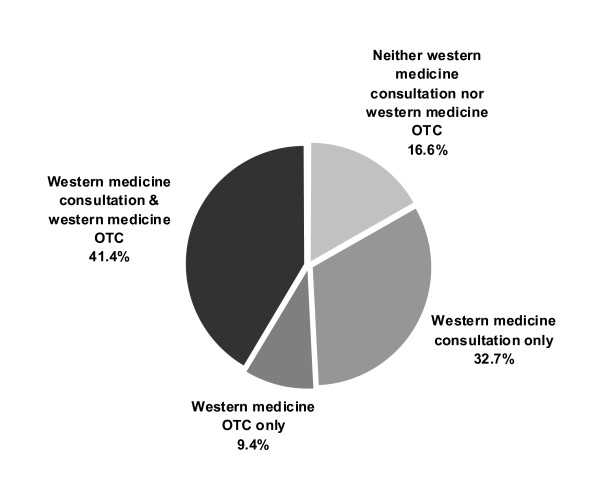
**Healthcare choices (western medicine) made by respondents in the previous year (*n *= 33,263)**.

Among all respondents (*n *= 33,263), 9.4% used western OTC medications only whereas 41.4% used both western medicine consultation services and western OTC medications in the previous year. 32.7% used western medicine consultation only, and 16.6% used neither western OTC medication nor western medical services (Figure 2). Univariate analysis indicated significant differences among the first three groups in terms of gender, age, education level, health status, chronic disease status, smoking habit, Chinese and/or western medicine insurance and income (Table 1). Multinomial logistic regression analysis (Table 2) showed that those who only used western OTC medications were more likely to be young adults to middle aged but not over 70, male, primary or secondary educated, having a lower personal monthly income and no insurance coverage for western medical services. They also self reported better perceived health status and being chronic disease free, being a smoker, and not exercising regularly. Those who used both western medicine consultation and western OTC medications demonstrated similar patterns with one exception that their incomes were higher.

**Table 1:** Demographic, socioeconomic and health related characteristics among western medical services and OTC medication users

		Visited western medicine practitioners only (%)	Used western OTC medications only (%)	Consulted western medicine practitioners and used western OTC medications (%)	*P *values ^a^
**Gender**	Male	38.2	12.5	49.3	< 0.001
	Female	39.6	9.9	50.5	
**Age**	15-29	36.6	12.0	51.4	
	30-39	34.3	11.8	53.9	< 0.001
	40-49	33.8	13.0	53.2	
	50-59	37.5	12.3	50.2	
	60-69	45.4	8.8	45.8	
	70+	58.6	4.4	37.0	
**Education level**	Below primary	54.1	5.7	40.2	
	Primary	39.7	11.5	48.7	< 0.001
	Secondary	34.9	12.6	52.5	
	Tertiary	43.6	8.4	48.1	
**Currently married**	Yes	38.3	11.2	50.5	
	No	40.0	11.0	49.0	
**Self reported health**	Excellent or very good	40.7	11.4	47.9	< 0.001
	Good or fair	37.5	11.5	51.1	
	poor	51.7	7.0	41.3	
**Self reported chronic disease status**	Yes	51.7	1.7	46.6	
	No	35.3	13.8	50.9	< 0.001
**Drinking habit**	Yes	37.0	13.7	49.3	
	No	39.2	10.8	50.1	
**Current smoker**	Yes	33.9	16.2	49.9	
	No	40.0	10.0	50.0	< 0.001
**Moderate exercise >=2.5 hours/week**	Yes	43.9	9.5	46.6	
	No	37.1	11.7	51.2	
**Possession of western medicine insurance**	Yes	38.2	7.5	54.3	
	No	39.2	12.4	48.5	< 0.001
**Possession of Chinese medicine insurance**	Yes	36.4	7.1	56.5	
	No	39.1	11.4	49.5	< 0.001
**Total**		**38.9**	**11.1**	**50.0**	
**Mean personal monthly income in Hong Kong dollars^b ^(Standard Error)**		$10042 ($148.64)	$8832 ($221.47)	$10117 ($121.21)	< 0.001

^a^: All from Chi square test for independence except for mean personal monthly income, in which one way ANOVA were used

^b^: HKD$ 7.8 = USD$ 1.0

**Table 2:** Association of demographic, socioeconomic and health related characteristics with choices for western medicine consultation and OTC medication

	Used western OTC medications only Adjusted Odds Ratio (95%CI)	P values	Choice between western medicine consultations and western OTC medications Adjusted Odds Ratios (95%CI)	P values
**Gender**				
Female	Reference		Reference	
Male	1.31 (1.17, 1.46)	< 0.001	1.03 (0.96, 1.10)	NS
**Age**				
15-29	Reference		Reference	
30-39	1.21 (1.02, 1.44)	0.032	1.09 (0.98, 1.22)	NS
40-49	1.28 (1.07, 1.53)	0.007	1.05 (0.94, 1.18)	NS
50-59	1.14 (0.93, 1.39)	NS	0.90 (0.79, 1.02)	NS
60-69	0.93 (0.73, 1.19)	NS	0.73 (0.63, 0.85)	< 0.001
70 or above	0.50 (0.38, 0.67)	< 0.001	0.49 (0.42, 0.57)	< 0.001
**Education level**				
Below Primary	Reference		Reference	
Primary	1.42 (1.09, 1.84)	0.008	1.15 (1.01, 1.32)	0.047
Secondary	1.35 (1.04, 1.75)	0.023	1.11 (0.97, 1.27)	NS
Tertiary	0.98 (0.72, 1.33)	NS	0.84 (0.71, 0.98)	0.027
**Marital status**				
Currently married	Reference		Reference	
Not currently married	1.00 (0.88, 1.13)	NS	0.95 (0.88, 1.03)	NS
**Self reported health**				
Poor	Reference		Reference	
Good/fair	1.44 (1.15, 1.80)	0.001	1.44 (1.28, 1.62)	< 0.001
Excellent/very good	1.21 (0.93, 1.57)	NS	1.19 (1.03, 1.38)	0.021
**Self reported chronic disease status**				
No	Reference		Reference:	
Yes	0.10 (0.08, 0.13)	< 0.001	0.82 (0.76, 0.89)	< 0.001
**Drinking habit**				
No	Reference		Reference	
Yes	1.03 (0.89, 1.21)	NS	0.97 (0.88, 1.07)	NS
**Current smoker**				
No	Reference		Reference	
Yes	1.56 (1.38, 1.78)	< 0.001	1.12 (1.02, 1.22)	0.010
**Moderate exercise >=2.5 hours/week**				
No	Reference		Reference	
Yes	0.81 (0.72, 0.91)	< 0.001	0.86 (0.80, 0.92)	< 0.001
**Possession of western medicine insurance (%)**				
No	Reference		Reference	
Yes	0.55 (0.47, 0.63)	< 0.001	0.99 (0.91, 1.07)	NS
**Possession of Chinese medicine insurance (%)**				
No	Reference		Reference	
Yes	0.91 (0.71, 1.15)	NS	1.12 (0.98, 1.27)	NS
**Monthly personal income** (for every increment of HK$ 1000, or US$ 128.2)	0.98 (0.98, 0.99)	< 0.001	1.00 (0.99, 1.00)	0.022

NS: Statistically non-significant (p > 0.05)

## Use of Chinese OTC medications and consultations with Chinese medicine practitioners

**Figure 3:**
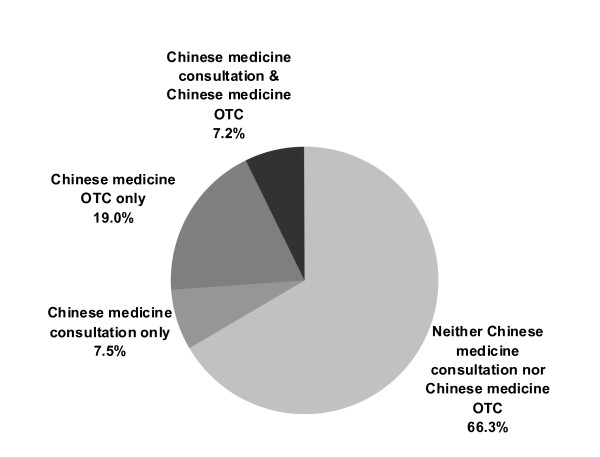
**Healthcare choices (Chinese medicine) made by respondents in the previous year (*n *= 33,263)**.

A total of 19.0% of the population used Chinese OTC medications only whereas 7.2% used both Chinese medicine consultation and Chinese OTC medication in the previous year. 7.5% used only Chinese medicine consultation; whereas 66.3% used neither Chinese OTC medication nor Chinese medicine consultation (Figure 3). Univariate analysis indicated significant differences among the first three groups in terms of gender, age, education level, self reported health status, chronic disease status, smoking, drinking, exercise habit, possession of Chinese and/or western medicine insurance coverage, and income (Table 3). Multinomial logistic regression analysis (Table 4) showed those who only used Chinese OTC medication were more likely to be aged 60 or above, male, to have received no formal education, to have a lower personal monthly income and no insurance coverage for TCM services. Also, they were more likely to report favourable perceived health status, to currently smoke, and to not exercise regularly. Those who used both Chinese medicine consultation and Chinese OTC medication were more likely to be middle aged, to have no insurance coverage for WMD services, and to suffer from chronic diseases.

**Table 3:** Demographic, socioeconomic and health related characteristics among Chinese medical services and OTC medication users

		Visited Chinese medicine practitioners only (%)	Used Chinese OTC medications only (%)	Consulted Chinese medicine practitioners and used Chinese OTC medications (%)	p-value^a^
**Gender**	Male	19.9	61.9	18.2	p < 0.001
	Female	23.9	51.7	24.4	
**Age**	15-29	26.9	57.3	15.8	p < 0.001
	30-39	29.4	50.8	19.7	
	40-49	24.1	52.4	23.5	
	50-59	21.9	56.0	22.2	
	60-69	16.5	57.6	25.9	
	70+	12.7	66.1	21.3	
**Education level**	Below primary	12.8	66.2	21.1	p < 0.001
	Primary	18.5	56.8	24.7	
	Secondary	22.8	56.5	20.6	
	Tertiary	34.1	44.6	21.2	
**Currently married**	Yes	22.1	55.5	22.4	
	No	22.2	57.5	20.3	
**Self reported health**	Excellent or very good	23.8	59.2	17.1	p < 0.001
	Good or fair	22.0	55.8	22.2	
	poor	22.0	55.4	22.6	
**Self reported chronic disease status**	Yes	17.3	57.8	24.9	p < 0.001
	No	23.8	55.5	20.6	
**Drinking habit**	Yes	22.6	55.6	21.8	p < 0.001
	No	22.1	56.2	21.7	
**Current smoker**	Yes	18.5	62.1	19.4	p < 0.001
	No	22.9	54.9	22.2	
**Moderate exercise >= 2.5 hours/week**	Yes	23.2	54.3	22.5	p < 0.001
	No	21.7	56.9	21.4	
**Possession of western medicine insurance**	Yes	30.5	48.8	20.8	p < 0.001
	No	19.7	58.3	22.0	
**Possession of Chinese medicine insurance**	Yes	38.6	36.1	25.4	p < 0.001
	No	20.8	57.7	21.4	
**Total**		**22.2**	**56.1**	**21.7**	
**Mean personal monthly income in Hong Kong dollars^b ^(Standard Error)**	$12060 ($350.59)	$8180 ($151.10)	$9305 ($306.72)		p < 0.001

^a^: All from Chi square test for independence except for mean personal monthly income, in which one way ANOVA were used

^b^: HKD$ 7.8 = USD$ 1.0

**Table 4:** Association of demographic, socioeconomic and health related characteristics with choices for Chinese medicine consultation and OTC medications

	Used Chinese OTC medications only Adjusted Odds Ratio (95%CI)	P values	Choice between Chinese medicine practitioner consultations and Chinese OTCmedications Adjusted Odds Ratios (95%CI)	P values
**Gender**				
Female	Reference		Reference	
Male	1.70 (1.49, 1.93)	< 0.001	0.94 (0.81, 1.10)	NS
**Age**				
15-29	Reference		Reference	
30-39	0.98 (0.79, 1.23)	NS	1.18 (0.89, 1.56)	NS
40-49	1.10 (0.87, 1.37)	NS	1.57 (1.19, 2.08)	0.002
50-59	1.18 (0.92, 1.49)	NS	1.49 (1.11, 2.01)	0.009
60-69	1.40 (1.06, 1.86)	0.020	2.08 (1.48, 2.92)	< 0.001
70 or above	1.75 (1.30, 2.35)	< 0.001	1.98 (1.38, 2.84)	< 0.001
**Education level**				
Below Primary	Reference		Reference	
Primary	0.66 (0.52, 0.85)	0.001	0.94 (0.71, 1.26)	NS
Secondary	0.69 (0.53, 0.89)	0.004	0.85 (0.64, 1.15)	NS
Tertiary	0.49 (0.36, 0.66)	< 0.001	0.72 (0.51, 1.03)	NS
**Marital status**				
Currently married	Reference		Reference	
Not currently married	1.09 (0.95, 1.26)	NS	1.06 (0.90, 1.26)	NS
**Self reported health**				
Poor	Reference		Reference	
Good/fair	1.38 (1.06, 1.81)	0.020	0.96 (0.70, 1.33)	NS
Excellent/very good	1.28 (1.04, 1.58)	0.022	1.22 (0.96, 1.55)	NS
**Self reported chronic disease status**				
No	Reference		Reference:	
Yes	1.05 (0.90, 1.23)	NS	1.28 (1.08, 1.53)	0.007
**Drinking habit**				
No	Reference		Reference	
Yes	0.86 (0.72, 1.04)	NS	1.06 (0.85, 1.31)	NS
**Current smoker**				
No	Reference		Reference	
Yes	1.20 (1.01, 1.42)	0.034	1.12 (0.92, 1.38)	NS
**Moderate exercise >= 2.5 hours/week**				
No	Reference		Reference	
Yes	0.82 (0.73, 0.93)	0.002	0.93 (0.80, 1.08)	NS
**Possession of western medicine insurance (%)**				
No	Reference		Reference	
Yes	0.87 (0.76, 1.01)	NS	0.83 (0.69, 0.99)	0.038
**Possession of Chinese medicine insurance (%)**				
No	Reference		Reference	
Yes	0.52 (0.42, 0.65)	< 0.001	0.93 (0.73, 1.19)	NS
**Monthly personal income** (for every increment of HK$ 1000, or US$ 128.2)	0.98 (0.98, 0.99)	< 0.001	1.00 (0.99, 1.00)	NS

NS: Statistically non-significant (p > 0.05)

## Discussion and recommendations

A total of 50.8% of the Hong Kong population used western OTC medication in the previous year. Assuming that the western OTC medication was obtained from western medicine community pharmacists, we contend that the role of community pharmacists in primary care must not be underestimated. This is vividly illustrated by the fact that 9.4% of the respondents had no consultation with western medicine practitioners in the previous year but depended on western OTC exclusively for their healthcare. This implies that for these individuals, western medicine community pharmacists might be the only point of contact when they had minor aliments. Therefore, there is a need in promoting and extending the roles of the western medicine community pharmacists in Hong Kong.

A systematic review shows that pharmacists are often perceived by laypersons as drug experts with limited knowledge on health issues, but customers are generally satisfied with their extended role in providing health advices [14]. In Hong Kong where the culture is unique, local populations' expectation on the extended roles of community pharmacists may be raised to improve patient-oriented community health services. Western medicine community pharmacists' self-perception as a primary care provider is also an important factor that determinant the success of their role extension. In Hong Kong, western medicine community pharmacists provide advice about medicines [15-18] rather than addressing the clients' wider determinants of health (only 44% of community pharmacists consider education activities as one of their main duties) [7]. Appropriate training may help community pharmacists make their primary care practice more evidence-based [19]. Furthermore, stronger incentives and support like such as remuneration should be considered [20].

Other factors such as proximity to other professionals, opportunity for inter-professional communication and access to patients' medical information are essential to integrate pharmacists in the primary care system [20]. The role of medical professionals is a dominant factor in defining, controlling and scoping the work of the allied health professionals [21,22] as extending pharmacists' role in primary care may affect the autonomy and control of the medical professionals [23], particularly the private western medicine practitioners who also dispense medications in their clinics [1]. A stronger linkage between community pharmacists and the primary care team should be established as 39% of pharmacists did not have frequent communication with other healthcare professionals [7]. A possible option for Hong Kong in the future would be the establishment of integrated prescribing and dispensing service by western medicine practitioners and pharmacists under the same roof in both private and public sectors but this would require much research and harmonization by the government. While this will pose a significant challenge for western medicine, the situation becomes even more complex when the integration with Chinese medicine is taken into account. Previous research has already indicated the needs for western pharmacists to study Chinese medicine [24].

A total of 26.2% of the respondents reported consuming Chinese OTC medication in the previous year. The majority of them (19%) reported having no consultation with a Chinese medicine practitioner within the same period. Chinese medicine retailers may have been the only source of guidance on for these respondents. Patients with chronic diseases on western medications are also likely to consume Chinese herbal medicines [25]. Chinese medicine retailers are therefore instrumental in preventing undesirable drug interactions during prescription process. In addition, their role and competence should goes beyond prescription and medication review. All those involved in providing pharmacy services, regardless of Chinese or western medical affiliations, should have a role in gate-keeping other medical services and in promoting health.

## Conclusion

We propose, in this article, an extended role for community pharmacists and Chinese medicine retailers, which entails aspects as follows: (1) referring patients to other medical services where appropriate; (2) providing health education and preventative services; (3) safeguarding the use of Chinese herbal medicines.

## Competing interests

The authors declare that they have no competing interests.

## Authors' contributions

VCHC, CHL and SMG conceived the research idea. CHL conducted the statistical analysis. VCHC interpreted the result and wrote the first draft of the manuscript. FWKC, JHSY, ELYW added critical comments on the interpretations of data and on the manuscript. SMG and EKY supervised the whole research process. All authors read and approved the final manuscript.
